# miR-492 promotes chemoresistance to CDDP and metastasis by targeting inhibiting DNMT3B and induces stemness in gastric cancer

**DOI:** 10.1042/BSR20194342

**Published:** 2020-03-09

**Authors:** Shuai Wu, Jian Xie, Hao Shi, Zi-wei Wang

**Affiliations:** 1 Department of Gastroenterological Surgery, the First Affiliated Hospital of Chongqing Medical University, Chongqing 400042, P.R. China; 2 Department of Gastroenterological Surgery, Yong chuan Hospital of Chongqing Medical University, Chongqing 400016, P.R. China

**Keywords:** Chemoresistance, DNMT3B, Gastric cancer, Metastasis, miR-492, Stemness

## Abstract

**Objective:** Metastasis and chemoresistance indicate treatment fail and progresses in gastric cancer (GC) patients. However, the molecular mechanisms of chemoresistance and metastasis remain unclear in GC. Thus, identifying the biological indicators of chemoresistance and metastasis is particularly important. **Materials and methods:** We establish a role for miR-492 in GC metastasis and chemoresistance through experiments *in vitro* and *in vivo*. **Results:** We identified miR-492 overexpression in GC specimens and cell lines, the miR-492 expression level was inversely correlated with the prognosis of GC patients. The inhibition of miR-492 suppressed GC cell invasion and enhanced the sensitivity of gastric cancer cells to CDDP treatment. In contrast, miR-492 overexpression significantly stimulated GC cell invasion and contributed to chemoresistance development. In addition, our research results indicated that the inhibition of miR-492 stimulates GC stemness, and the overexpression of miR-492 induces GC stemness. Importantly, our experiments demonstrated that miR-492 inhibitor suppressed tumor formation, and the combination treatment of miR-492 inhibitor and CDDP significantly inhibited tumor growth *in vivo*. Furthermore, we demonstrated that miR-492 exerts its anticancer role by targeting DNMT3B in GC. **Conclusions**: Our results suggested that inhibiting miR-492 is a novel strategy to control tumor metastasis and chemoresistance in GC.

## Introduction

Gastric cancer (GC) is a common tumor of the digestive system [1]. Almost two-thirds of the cases occur in developing countries and 42% in China alone [2]. Metastasis and chemoresistance indicate treatment fail and progress in gastric cancer (GC) patients. However, chemoresistance and metastasis are still the main challenges in the treatment of GC patients. However, the molecular mechanisms of chemoresistance and metastasis remain unclear in GC. Thus, identifying the biological indicators of chemoresistance and metastasis is particularly important.

Cancer stem cells (CSCs) are a small subset of cells within tumor, and studies show that CSCs are implicated in chemotherapy resistance and metastasis in cancers [3]. CSCs present high resistance to chemotherapy drugs that are commonly used in the treatment of GC, including cisplatin [4,5]. Some studies showed that CSCs are able to regenerate all of the cell types in tumors because they have stem cell-like behavior that leads to metastatic recurrence [4,6]. Therefore, CSCs are important therapeutic targets in cancer. However, the mechanism of CSCs regulation in GC remains unclear.

Dysregulated expression of miRNAs was detected in most cancer types, and dysregulation of even a single miRNA can lead to tumorigenesis and stimulate cancer progression [7,8]. Additionally, dysregulated expression of miRNAs was demonstrated in CSCs, and such aberrantly regulated miRNAs participate in the development of CSCs and maintenance of stemness [9]. Previous studies showed that MiR-492 can regulate metastatic properties of hepatoblastoma via CD44, which is also a stemness protein marker. However, its function and mechanism in GC remain unclear. So, we want to study whether miR-492 will affect the progression of gastric cancer through stemness. Here, we describe the functional role of miR-492 as a tumor promoter in regulating metastasis, chemoresistance and CSCs. Additionally, we identified DNMT3B as a target of miR-492 in GC. We also found that miR-492 is significantly overexpression in GC and participates in stemness phenotypic via post-transcriptional regulation of DNMT3b.

## Materials and methods

### Cell and human specimens’ information

All GC cells (AGS and SGC7901) were purchased from the ATCC (Manassas, VA). RPMI 1640 media (Sigma-Aldrich) and fetal bovine serum (HyClone) were used in the present study. Human specimens were obtained from diagnostic biopsies. A total of 40 diagnostic patient specimens and adjacent tissue were used (Table 1), and informed consent was obtained from each patient who participated in the present study.

**Table 1:** Characteristics of gastric carcinoma patients

Characteristics	Variable	Number	*P* value
Age (years)	Range, (mean ± SD)	35–77 (60 ± 12)	0.189
Gender	Male	24 (63.3)	0.952
	Female	16 (36.7)	
Family history	No	29 (80.0)	0.424
	Yes	11 (20.0)	
Clinic stage	I	4 (6.7)	0.315
	II	14 (36.7)	
	III	13 (33.3)	
	IV	9 (23.3)	
Pathological type	Intestinal type	28 (80.0)	0.837
	Diffuse type	22 (13.3)	

### qRT-PCR analysis

TRIzol reagent (Invitrogen, Carlsbad, CA) were used to RNA extracted and subjected to qRT-PCR. The miR-492 and RNU6 expression was measured by qRT-PCR using the primer set from RiboBio (Guangzhou, China). The primer sequences were defined in Table 2.

**Table 2:** The primer sequences information

Pyrosequencing primers	Sequence (5′-3′)
DNMT3B Forward	ATGTAATGCTCCCCTCACCC
DNMT3B Reverse	CCTGAATCTGGGGCATGGTA
GAPDH Forward	GCAGGGGGGAGCCAAAAGGGT
GAPDH Reverse	TGGGTGGCAGTGATGGCATGG
DNMT3B (3′ UTR) Forward	GCTCTAGACAGCCAGGCCCCAAGCCC
DNMT3B (3′ UTR) Reverse	GCTCTAGAACCTCAGGCTACCCCTGC
Nanog Reverse 1	CCTACATAATAACATAAAACAACCAACTCA
Nanog Forward 1	[Bio]AAGTATTTGTTGTTGGGTTTGTTTTTAGG
Nanog Reverse 2	[Bio]AAAATAACTACAAAATAACCCAAACTAAAT
Nanog Forward 2	TTTTTAATTTATTGGGATTATAGGGGTGGG
OCT-3/4 Reverse 1	[Bio]CCCCATCRAAATTACTCTCCACCC
OCT-3/4 Forward 2	TTGGGTTAGGTTTTGAGGTGT
OCT-3/4 Reverse 1	[Bio]CCATCAAACTACCCTATCATAACC
OCT-3/4 Forward 2	TGGAGTGGGGTTAGTGTT

### Immunofluorescence

The immunofluorescence assays were performed as described by Roscigno et al. [10].

### Cell viability assay

Cells were transfected with the indicated plasmids. Twenty-four hours after transfection, cell proliferation was evaluated using cell viability assays with a Cell Counting Kit-8 (CCK-8, Med Chem Express, Monmouth Junction, New Jersey, U.S.A.).

### Apoptosis analysis

The cells were seeded in 6-well cell culture plates. Cells were incubated with the CDDP (10 μg/ml). After 24 h, cells were stained with annexin V and 7-aminoactinomycin D (7-AAD) according to the manufacturer’s protocol (Biotium Inc., Fremont, CA). The cells were then analyzed using flow cytometry.

### CD133 flow detection

Wash the transplanted tumor tissue in a sterilized dish, wash it with hanks buffer, and cut it into pieces of tissue about 3 mm^3^; Add 3 ml serum-free 1640 medium to the culture dish, and then add the tissue fragments Piece. Then add 3 ml collagenase (0.1%); put the Petri dish into the incubator for 3 h for digestion; blow the tissue fragments and pass through a 70 μm cell sieve to make a cell suspension (Cell testing starts from this step); suspend cells and centrifuge the solution for 10 min at 1200 rpm, remove the supernatant, and wash once with pbs; adjust the four groups of cells obtained to 1 × 10^6^ per 100 μl and transfer to EP tubes. Add 1 μl FC block antibody to each tube and incubate for 30 min, wash once with PBS. Add 5 μg of CD133-FITC antibody (abcam, U.S.A.) to each tube, and protect from ice for 30 min in the dark; add 2 ml of cell staining buffer, centrifuge at 350 ***g*** for 5 min, remove the supernatant, and repeat twice; Resuspend the cells in 0.4 ml cell staining buffer, add 2 μl million cells of nuclear dye 7-AAD, and incubate on ice for 3–5 min; the cells were then analyzed using flow cytometry.

### Transwell and osteosphere assays

Transwell and osteosphere assays were performed according to the description of Xu et al. and Roscigno et al. [4,10].

### Luciferase reporter assay

The luciferase reporter assays were performed according to the description of Roscigno et al. [10].

### DNA methylation analysis by pyrosequencing

DNA methylation analyses were performed as described by Roscigno et al. [10]. The primer sequence information in Table 2.

### Western blot

About 35 µg protein was separated by sodium dodecyl sulfate-polyacrylamide gel electrophoresis and transferred onto nitrocellulose membranes. All antibodies used in the present study were purchased from Abcam (Cambridge, MA).

### Animal experiments

About 1 × 10^6^ SGC7901 CDDP resistance cells in 100 µl serum-free medium, which stably transfected with a series of miR-492 or negative control lentiviruses were constructed in our laboratory, were injected subcutaneously (s.c.) into per mouse (right back). When the tumors reached ∼50 mm^3^, which were intraperitoneally (i.p.) injected with CDDP (6 mg/kg). Following implantation, tumor volumes and body weight were measured every 7 days until the mice were killed by CO_2_ at day 30 (no anesthetics used in this experiments). Six-week-old female athymic (nu/nu) mice were used in these experiments and 8 mice each group. Mice were housed under specific pathogen free conditions and the veterinarian monitor the health and behavior of animal everyday morning. All animal experiments were performed in Central Laboratory of Yongchuan Hospital, Chongqing Medical University and were approved by ethics committee of the Yongchuan Hospital of Chongqing Medical University (2019055).

### Statistical analysis

All statistical analysis using the GraphPad Prism 8 software. *P* < 0.05 was considered significant. Statistical significance was analysed by unpaired Student’s *t* tests or one-way ANOVA and Duncan’s multiple range tests. Kaplan–Meier survival analysis was used to calculate the overall survival rate of gastric cancer.

## Results

### miR-492 expression was associated with poor clinical outcome

**Figure 1:**
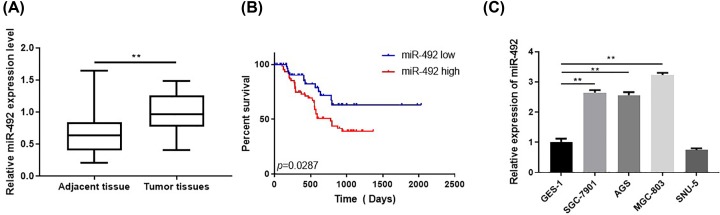
The expression of miR-492 was associated with gastric cancer’s outcome (**A**) The data demonstrated that the expression of miR-492 were down-regulated in GC specimens and overexpression in normal gastric tissues. (**B**) The clinical data showed that decreased expression of miR-492 was significantly correlated with poor overall survival and that overexpression of miR-492 was significantly correlated with good outcome in GC patients. (**C**) Relative expressions of miR-492 in gastric cancer cell lines and normal cell line; **P < 0.01.

The data demonstrated that compared with normal gastric tissues miR-492 expression was significantly increased in GC specimens (Figure 1A). Our data found that miR-492 was associated with clinical poor outcomes in GC patients (Figure 1B). Consistent with these clinical data, the miR-492 expression was decreased in GC cell lines compared with the Human gastric mucosal cells GES-1 (Figure 1C).

### MiR-492 induces proliferation and metastasis in gastric cancer cells

**Figure 2:**
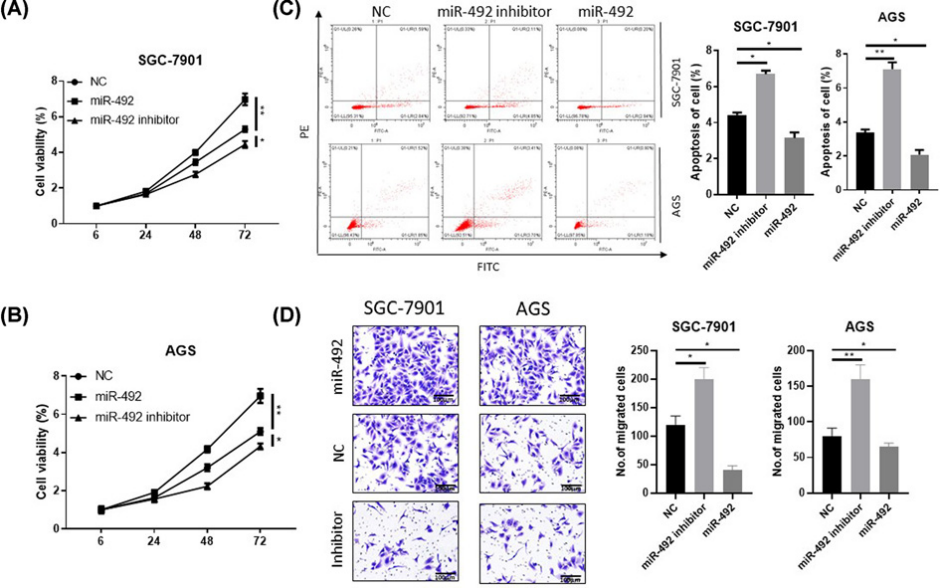
MiR-492 suppress the proliferation and invasion in gastric cancer cells (**A** and **B**) the CCK-8 assays investigated the effects of miR-492 on GC proliferation using two GC cell lines (SGC7901 and AGS) transfected with miR-492 mimic or inhibitors. (**C**) Flow cytometric analysis of apoptosis in miR-492 over-expression or knock-down in SGC7901 and AGS cell lines. (**D**) Transwell invasion assay of miR-492 over-expression or knock-down in SGC7901 and AGS cell lines; *P < 0.05, **P < 0.01.

The Figure 1 showed that miR-492 was associated with clinical poor outcomes in GC patients and that the rapid tumor growth and occurrence of metastasis and indicate poor clinical outcomes in GC patients. Thus, we investigated the effects of miR-492 on GC metastasis and proliferation using two GC cell lines via up or down-regulating miR-492 (Supplementary Figure S1). The cell viability was increased in miR-492-overexpressing cells, but the cell viability was decreased in miR-492-inhibit cells by CCK-8 assays (Figure 2A,B). And then, apoptosis analysis results show that ectopic miR-492 expression suppressed GC cell apoptosis and that the inhibition of miR-492 stimulated GC cell apoptosis compared with control group (Figure 2C). In addition, transwell experiments showed that the miR-492 overexpression promoted GC cell metastasis, while the inhibition of miR-492 inhibited GC cell invasion (Figure 2D). Together, our above data suggest that miR-492 promoted GC progression by inducing GC cell invasion and proliferation, suppressed the apoptosis.

### miR-492 induces CSCs in GC

**Figure 3:**
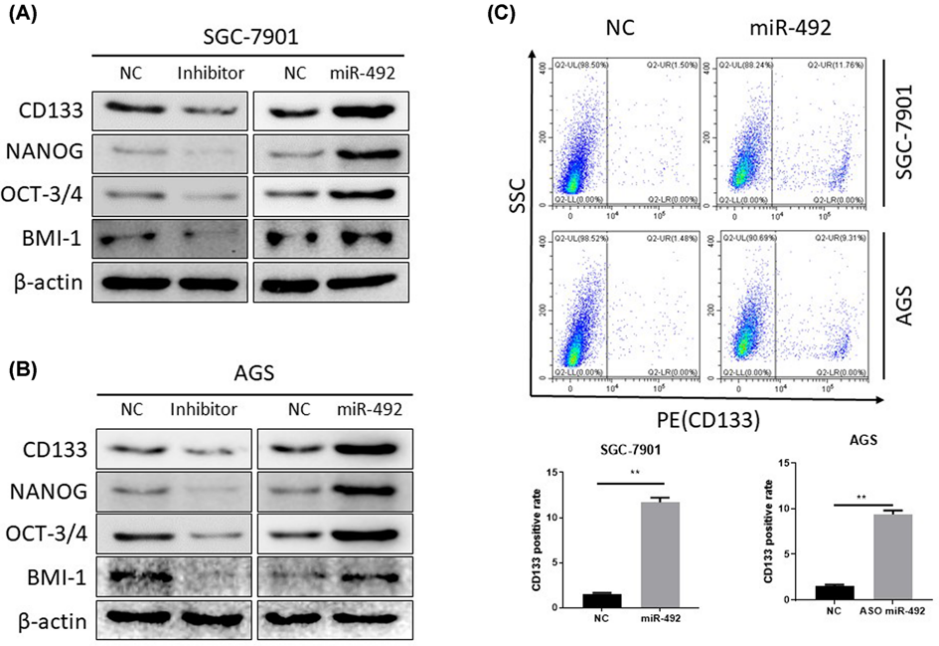
MiR-492 can inhibit CSCs in gastric cancer cells (**A** and **B**) The overexpression of miR-492 up-regulated the CSCs marker protein (CD133, SOX2, OCT4 and BMI-1) expression, but the inhibition of miR-492 negatively regulated CSCs marker protein (CD133, Nanog, OCT-3/4 and BMI-1) expression. SGC7901 and AGS cells were transfected with the indicated nucleotides. After 72 h of transfection, cells were subjected to Western blot analysis. (**C**) The flow cytometric analysis showed that inhibition of the miR-492 expression could induce the expression of stemness-related protein CD133. NC, negative control oligonucleotides; mimics: miR-492 mimics; inhibitor, miR-492 inhibitor; **P < 0.01.

Because previous studies have shown that CSCs cause progression and metastasis in cancers, we investigated whether miR-492 is involved in CSCs regulation of GC. The Western blot results found that the miR-492 overexpression significantly up-regulated CSCs marker proteins expression, including CD133, Nanog, OCT-3/4 and BMI-1 in two cells (Figure 3A,B). The inhibition of miR-492 expression suppressed these CSCs marker proteins expression (Figure 3A,B). The flow cytometric assay showed that inhibiting the miR-492 expression could induce the expression of CD133, a stemness-related protein. These data suggested that miR-492 exerts its tumor promoting effect partially due to the induction of CSCs in GC (Figure 3C).

### miR-492 target DNMT3B and suppress its expression

**Figure 4:**
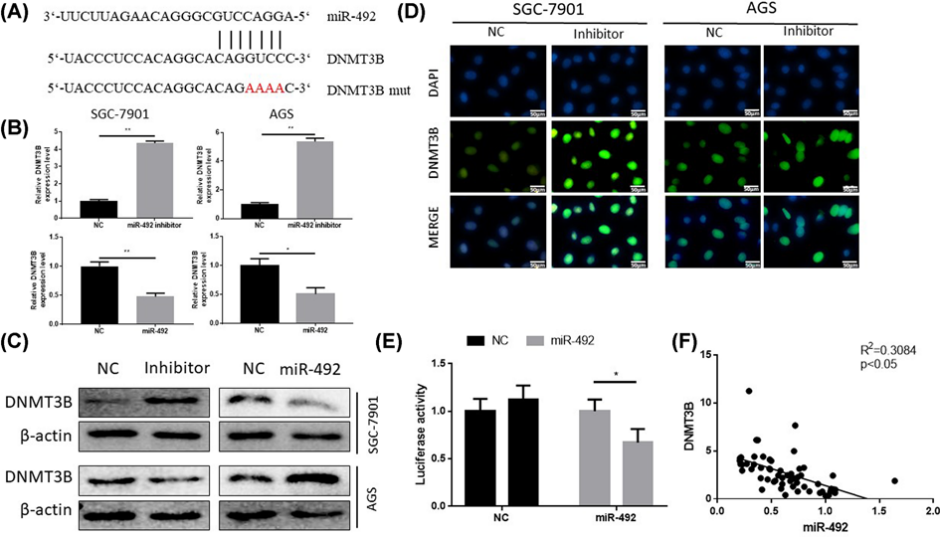
DNMT3B is a target of miR-492 (**A**) The miR-492 seed sequence is complementary to the 3′ UTR of DNMT3B. (**B**) MiR-miR-492 inhibited DNMT3B mRNA expression in both SGC7901 and AGS cell lines. After 72 h of transfection, the expression of DNMT3B was measured using qRT-PCR. (**C** and **D**) miR-492 inhibited DNMT3B protein expression. SGC7901 and AGS cells were transfected with the mimics and the inhibitors get the opposite result. After 72 h of transfection, the expression of DNMT3B was measured using Western blot and immunofluorescence. (**E**) Activity of the luciferase gene linked to the 3′ UTR of DNMT3B. The luciferase reporter plasmids of wild-type (WT) or mutated 3′ UTR sequences of DNMT3B (MT) were transfected into HEK-293 cells with or without the miR-492 mimic. (**F**) The expression levels of DNMT3B and miR-42 showed a negative correlation in GC patients that were measured by RT-qPCR. Tumor samples were obtained from 60 patients with GC. NC, negative control oligonucleotides; mimic, miR-492 mimic; inhibitor, miR-492 inhibitor; ns, no significance. *P < 0.05, ** *P* < 0.01.

Further, to investigate how miR-492 regulates CSCs in GC, we searched for target gene candidates of miR-492 (mirdb.org) and identified DNMT3B as a candidate of miR-492 (Figure 4A). DNMT3B is involved in cancer stemness maintenance and is closely associated with cancer proliferation and metastasis [10]. So, we chose to further study DNMT3B. To investigate whether miR-492 regulated DNMT3B, we examined DNMT3B expression levels by Western blot. Our experiment results showed that DNMT3B expression was up-regulated or down-regulated by the inhibition or ectopic expression of miR-492, respectively, in GC cells at both the mRNA and protein levels (Figure 4B,C). The immunofluorescence also confirmed this result (Figure 4D). We verified that miR-492 directly targeted the 3′ UTR of DNMT3B by luciferase reporter assay (Figure 4E). Furthermore, consistent with the cell results, the clinical sample analysis results also showed an inverse association between DNMT3B and miR-492 in GC specimens (Figure 4F).

### MiR-492 inhibitor induces the apoptosis and suppresses the metastasis by target DNMT3B

**Figure 5:**
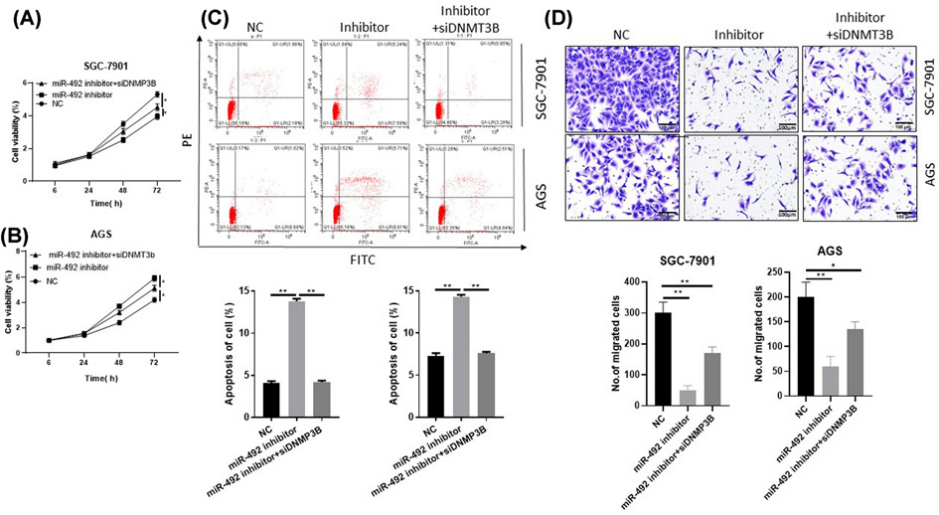
MiR-492 inhibitor induces the apoptosis and suppresses the metastasis by target DNMT3B (**A** and **B**) The CCK-8 assays showed that gastric cancer cell proliferation was decreased in the miR-492 inhibitor group, but it was restored in the siDNMT3B group compared with the miR-492 inhibitor group. (**C**) The down-regulation of DNMT3B restored the apoptosis rate of cells suppressed by miR-492 inhibitor. The apoptosis assay analysis by flow cytometric in miR-492 inhibitor and/or siDNMT3B transfected SGC7901 and AGS cells. (**D**) The transwell invasion assay in miR-492 inhibitor and DNMT3B siRNA transfected GC cells. knockdown DNMT3B could reverse the suppression of metastasis by miR-492 inhibitor; *P < 0.05, ** *P <* 0.01.

We next transfected mimics specific for miR-492 inhibitor and siDNMT3B plasmid AGS and SGC-7901 cells. The CCK-8 assays showed that gastric cancer cell proliferation was decreased in the miR-492 inhibitor group, but it was restored in the siDNMT3B group compared with the miR-492 inhibitor group (Figure 5A,B). The cell apoptosis rate was higher when miR-492 was suppressed, but lower when combined with siDNMT3B (Figure 5C). Consistent with the apoptosis analysis, knockdown DNMT3B could reverse the suppression of metastasis by miR-492 inhibitor (Figure 5D). Together, our data suggest that miR-492 overexpression promoted cancer progression in gastric cancer.

### miR-492 target DNMT3B modulates GC stemness

**Figure 6:**
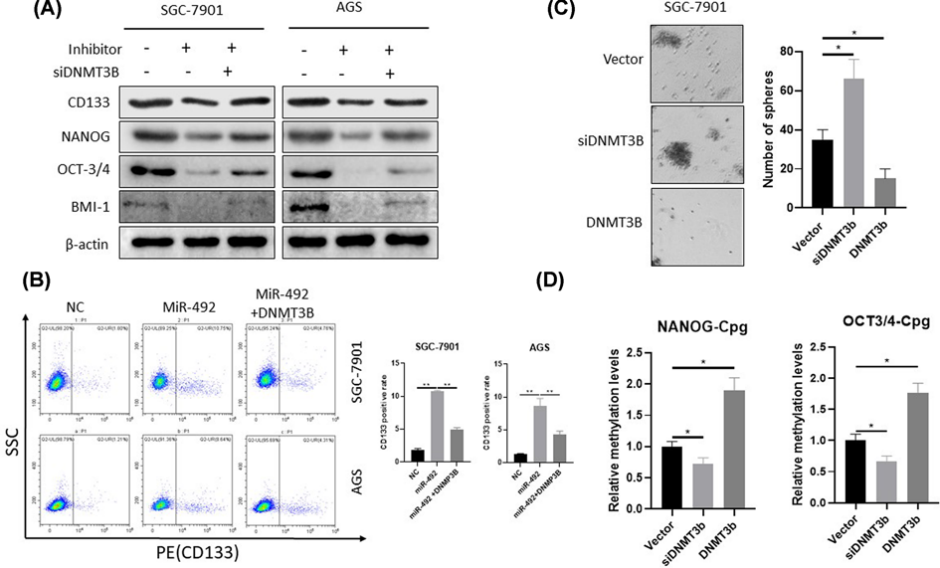
MiR-492 target DNMT3B that modulates GC stemness (**A**) The knockdown of DNMT3B restored the down-regulated expression of CSCs marker proteins inhibited by miR-492 inhibitor, including CD133, Nanog, OCT-3/4 and BMI-1. SGC7901 and AGS cells transfected with miR-492 inhibitor and/or siDNMT3B were subjected to Western blot analysis for the indicated proteins. (**B**) The overexpression of DNMT3B blocked the miR-492 mimics induce of CSCs marker CD133 protein expression. SGC7901 and AGS cells transfected with miR-492 mimic and/or DNMT3B expression plasmids were subjected to the flow cytometric analysis for the CD133. (**C**) The overexpression of DNMT3B stimulated osteosphere formation, while silencing of DNMT3B attenuated osteosphere formation, SGC7901 cells transfected with DNMT3B siRNA or DNMT3B expression plasmids were subjected to the osteosphere assays. (**D**) The pyrosequencing analysis revealed that cells transfected with a siDNMT3b showed a significant decrease (28% and 33%, respectively) in methylation levels at CpGs analyzed on Nanog and Oct 3/4 promoters compared with the scrambled control cells, but the DNMT3b plasmid transfected cells showed a significant increase (89% and 77%, respectively) in methylation levels at CpGs analyzed on Nanog and Oct 3/4 promoters NC, negative control oligonucleotides; mimic, miR-492 mimic; siDNMT3b: DNMT3b siRNA. *, *P* < 0.05; **, *P* < 0.01.

As we know, DNMT3b is a major regulator of Nanog and Oct 3/4 expression and inhibits their expression during embryogenesis through their methylation activity [10]. Therefore, to investigate whether miR-492-mediated regulation of CSCs by DNMT3B in GC. Our Western blot showed that the knockdown of DNMT3B restored the down-regulated expression of CSCs marker proteins inhibited by miR-492 inhibitor, including CD133, Nanog, OCT-3/4 and BMI-1 (Figure 6A). In contrast, up-regulation of DNMT3B blocked miR-492-induced expression of CD133 (a CSCs marker protein) (Figure 6B). Osteosphere formation results in the overexpression of DNMT3B-stimulated osteosphere formation, while silencing of DNMT3B attenuated osteosphere formation (Figure 6C). Then, pyrosequencing analysis showed that cells transfected with siDNMT3b showed significantly lower levels of CpGs methylation at the Nanog and Oct 3/4 promoters (28% and 33%, respectively) compared with control cells, but DNMT3b significantly increased methylation levels of CpG analyzed on Nanog and Oct 3/4 promoters (89% and 77%, respectively) (Figure 6D). Our results suggest that miR-492 regulates CSCs due to DNMT3B in GC.

### Down-regulated miR-492 reverses chemoresistance of CDDP via target DNMT3B *in vitro*

**Figure 7:**
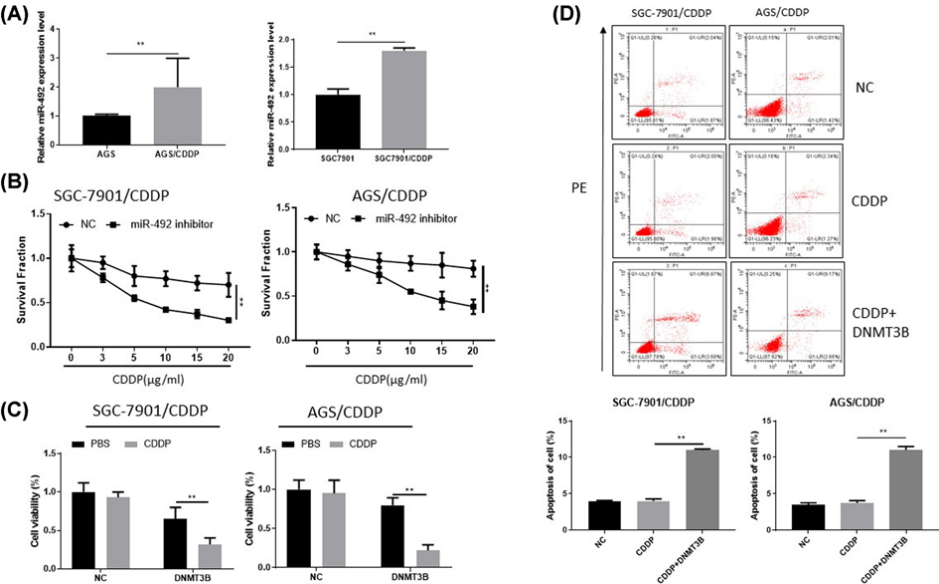
MiR-492 target DNMT3B reverse chemoresistance of CDDP *in vitro* (**A**) RT-PCR examined the expression of miR-492 in both SGC7901, SGC7901 (CDDP resistance) and AGS, AGS (CDDP resistance) cell lines via, showed that miR-492 overexpression in the CDDP resistance cell lines. (**B**) MiR-492 inhibitors could suppress cisplatin killing of gastric cancer cells, CCK-8 proliferation assay of miR-492 inhibitors transfected in SGC7901 and AGS cells. (**C**) Overexpression DNMT3B by DNMT3B plasmid could promote cisplatin killing of gastric cancer CDDP resistance cells, CCK-8 proliferation assay of DNMT3B transfected in SGC7901 and AGS cisplatin resistance cells. (**D**) Overexpression of DNMT3B combine CDDP could promote apoptosis rate of gastric cancer cisplatin resistance cells, DNMT3B plasmid transfected in SGC7901 and AGS cisplatin resistance cells, apoptosis assay analysis by flow cytometric assay; ** *P <* 0.01.

The CSCs closely related to cancer chemoresistance. Therefore, we investigated whether down-regulation of miR-492 could promote cisplatin killing of gastric cancer. We examined the miR-492 expression in both SGC7901/SGC7901 CDDP resistance and AGS/AGS CDDP resistance cell lines via RT-PCR, and the data showed that miR-492 overexpression in the CDDP resistance cell lines (Figure 7A). And then, the CCK-8 proliferation assay of miR-492 inhibitors transfected in SGC7901CDDP and AGSCDDP cells, knockdown miR-492 could increase promote cisplatin killing of gastric cancer cisplatin resistance cells (Figure 7B). Overexpression of DNMT3B by DNMT3B plasmid could promote cisplatin killing of gastric cancer cisplatin resistance cells (Figure 7C). Overexpression of DNMT3B that combine CDDP (5 µg/ml) could promote apoptosis rate of gastric cancer cisplatin resistance cells, the apoptosis assay analysis by flow cytometric assay (Figure 7D).

### MiR-492 inhibitor significantly inhibits tumorigenesis and chemoresistance of CDDP *in vivo*

**Figure 8:**
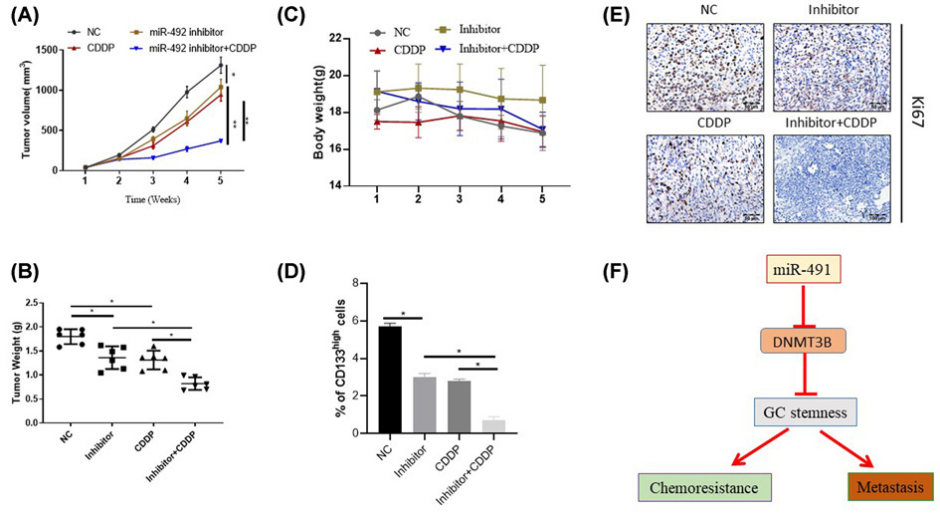
MiR-492 inhibitor reverse chemoresistance of CDDP *in vivo* (**A** and **B**) The tumor volume and tumor weight were significantly decreased when miR-492 inhibitor combine with CDDP compared with the other groups. (**C**) The body weight was not significantly different when miR-492 inhibitor combine with CDDP compare to the other groups The number of CD133^high^ were significantly lower in the miR-492 inhibitor combine CDDP treatment group compared to the control and miR-492 inhibitor and cisplatin alone treatment group. The SGC7901 CDDP resistance xenograft cells were subjected to the flow cytometric analysis for the CD133. (**D** and **E**) The IHC analyze the expression levels of Ki67 were significantly lower in the miR-492 inhibitor combine cisplatin treatment group compared to the control. (**F**) Graphic summary of the present study, the overexpression of miR-492 dramatically induce the CDDP chemoresistance of GC and promote GC metastasis through induce GC stemness by targeting DNMT3B; *P < 0.05, ** *P <* 0.01.

Then, we investigated the effects of miR-492 inhibitor on tumorigenesis and chemoresistance *in vivo* (Figure 8). As shown in (Figure 8A,B) tumor volume and tumor weight were significantly decreased when miR-492 inhibitor combine with CDDP compared with the other groups. Silencing of miR-492 expression combine with CDDP could decrease the percent of CD133^high^ cells in xenograft (Figure 8C). As expected, the expression levels of Ki67 were lower in the miR-492 mimics combine with cisplatin treatment group compared with the control (Figure 8D). Taken together, the silence of miR-492 dramatically restored the resistance of GC cells to chemotherapy, and inhibited GC metastasis through suppressing GC stemness by targeting DNMT3B.

## Discussion

The chemoresistance and metastasis in GC patients indicate poor outcome [11]. We used a series of experiments to study the effects of miR-492 on metastasis and chemoresistance in GC. Here, we found that silence of miR-492 expression significantly correlated with good clinical outcomes. In addition, our study showed that miR-492 inhibitor suppressed GC cell invasion and chemoresistance *in vitro*. Furthermore, the knockdown of miR-492 enhanced the chemosensitivity of GC cells to CDDP.

Next, we clarified the mechanism of miR-492 promote chemoresistance and metastasis. Accumulated evidence has shown that increased cancer stemness can stimulate cancer metastasis and induce chemoresistance [12–14]. Our data showed that miR-492 overexpression stimulated GC stemness. More importantly, our experiments showed that the silence of miR-492 significantly suppressed GC stemness populations in tumor tissues, suggesting that miR-492 plays an promote cancer role partially due to the induce of GC CSCs. In addition, we investigated the mechanism of miR-492 regulate CSCs in GC. We identify DNMT3B as a target gene of miR-492 in GC. Previous studies have shown that DNMT3b is a regulator of Nanog and Oct 3/4 expression and inhibits their expression during embryogenesis through their methylation activity [15]. DNMT3B is a CSCs marker and involved in CSCs regulation. Our data showed that DNMT3B expression was increased or decreased in GC cells by the inhibition or ectopic expression of miR-492, respectively. miR-492 directly targets the 3′ UTR of DNMT3B. Furthermore, the expression levels of miR-492 and DNMT3B were inversely correlated in GC patient specimens. Additionally, restore the DNMT3B could block the miR-492 overexpression-induced promotion of CSCs. Taken together, miR-492 promotes GC metastasis and chemoresistance through the stimulation of CSCs by targeting DNMT3B.

In summary, we establish a role for miR-492 in GC metastasis and chemoresistance through experiments. The inhibition of miR-492 dramatically enhances the sensitivity of GC cells to CDDP chemotherapy and inhibits GC metastasis through suppressing GC stemness by targeting DNMT3B. Our findings may also help develop potential therapeutics for the GC.

## Supplementary Material

Supplementary Figure S1

## Data Availability

The data sets used and/or analyzed during the present study are available from the corresponding author on reasonable request.

## Abbreviations

7-AAD: 7-aminoactinomycin D
CSC: cancer stem cell
GC: gastric cancer
