# Supplementary material for: miR-492 promotes chemoresistance to CDDP and metastasis by targeting inhibiting DNMT3B and induces stemness in gastric cancer
Source: Biosci Rep. 2020 Mar 9;40(3):BSR20194342. doi: 10.1042/BSR20194342 (PMC7064790; doi:10.1042/BSR20194342)
Supplement: Supplementary Figure S1 [file BSR-2019-4342_supp.pdf]

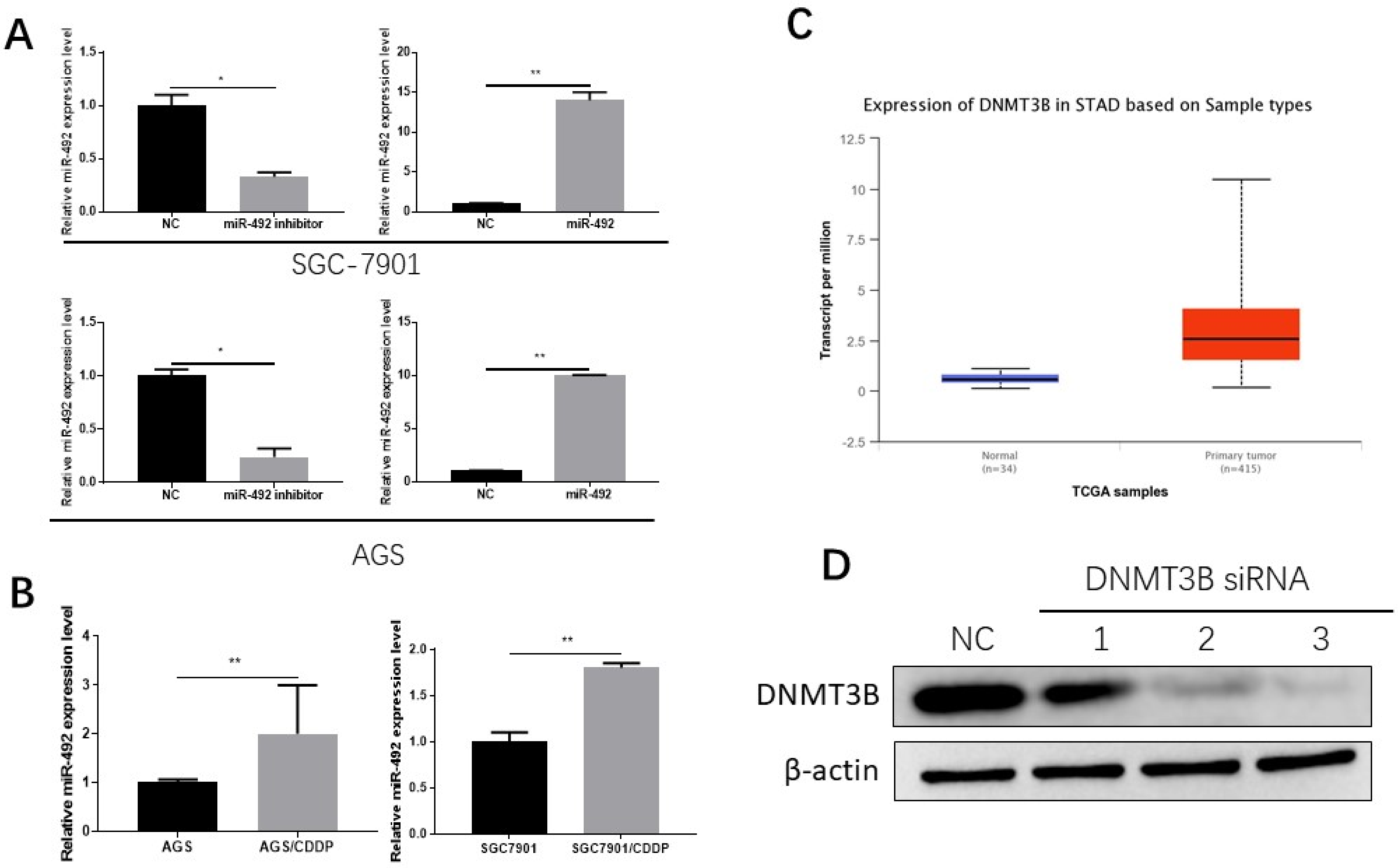

Supplement fig1

Supplemental data

Figure S1 **A:** SGC7901 and AGS cells were transfected with negative control oligonucleotide, miR-492 mimics or miR-492 inhibitor. After 72 hours of transfection, isolated mRNAs were subjected to qRT-PCR. **B**: The expression of miR-492 in SGC7901, SGC7901 (CDDP resistance) and AGS, AGS (CDDP resistance) cell lines were subjected to qRT-PCR. **C**: The analysis of DNMT3B expression by TCGA database. **D**: The siRNA knocks down DNMT3B detected by western blotting. *$P<0.05$, ** $P<0.01$.
